# Effects of flattening filter‐free and volumetric‐modulated arc therapy delivery on treatment efficiency

**DOI:** 10.1120/jacmp.v14i6.4328

**Published:** 2013-11-04

**Authors:** Evan M. Thomas, Richard A. Popple, Brendan M. Prendergast, Grant M. Clark, Michael C. Dobelbower, John B. Fiveash

**Affiliations:** ^1^ Department of Radiation Oncology University of Alabama at Birmingham Birmingham AL USA

**Keywords:** flattening filter free, treatment time, TrueBeam, IMRT, VMAT, RapidArc

## Abstract

Flattening filter‐free (FFF) beams are available on an increasing number of commercial linear accelerators. FFF beams have higher dose rates than flattened beams of equivalent energy which can lead to increased efficiency of treatment delivery, especially in conjunction with increased FFF beam energy and arc‐based delivery configurations. The purpose of this study is to quantify and assess the implications of improved treatment efficiency for several FFF delivery options on common types of linac applicable radiotherapy. Eleven characteristic cases representative of a variety of clinical treatment sites and prescription doses were selected from our patient population. Treatment plans were generated for a Varian TrueBeam linear accelerator. For each case, a reference plan was created using DMLC IMRT with 6 MV flat beams. From the same initial objectives, plans were generated using DMLC IMRT and volumetric‐modulated arc therapy (VMAT) with 6 MV FFF and 10 MV FFF beams (max. dose rates of 1400 and 2400 MU/min, respectively). The plans were delivered to a phantom; beam‐on time, total treatment delivery time, monitor units (MUs), and integral dose were recorded. For plans with low dose fractionations (1.8–2.0 & 3.85 Gy/fraction), mean beam‐on time difference between reference plan and most efficient FFF plan was 0.56 min (41.09% decrease); mean treatment delivery time difference between the reference plan and most efficient FFF plan was 1.54 min (range: 0.31–3.56 min), a relative improvement of 46.1% (range: 29.2%‐59.2%). For plans with high dose fractionations (16–20 Gy/fraction), mean beam‐on time difference was 6.79 min (74.9% decrease); mean treatment delivery time difference was 8.99 min (range: 5.40–13.05 min), a relative improvement of 71.1% (range: 53.4%‐82.4%). 10 MV FFF VMAT beams generated the most efficient plan, except in the spine SBRT case. The distribution of monitor unit counts did not vary by plan type. In cases where respiratory motion management would be applicable, 10 MV FFF DMLC IMRT reduced beam‐on time/field to less than 12 sec. FFF beams significantly reduced treatment delivery time. For radiosurgical doses, the efficiency improvement for FFF beams was clinically significant. For conventional fractionation, a large improvement in relative treatment delivery time was observed, but the absolute time savings were not likely to be of clinical value. In cases that benefit from respiratory motion management, beamon/field was reduced to a time for which most patients can comfortably maintain deep inspiratory breath hold.

PACS numbers: 87.55.D‐, 87.55.de, 87.56.bd, 87.56.N‐

## I. INTRODUCTION

Flattening filters were incorporated in linear accelerator beam lines to increase the dose homogeneity,^(1)^ which was important for minimizing normal tissue toxicity.^(2)^ As the clinical use of cranial radiosurgery and extracranial stereotactic body radiation therapy (SBRT) has increased, there has been increasing interest in the use of flattening filter‐free (FFF) beams to decrease treatment time and reduce scatter.^(^ ^3^ ^,^ ^4^ ^)^ Decreased treatment time is particularly meaningful if it can reduce the duration of treatment time slots, improve the patient's treatment experience, or reduce the likelihood of intrafraction motion. If dose homogeneity is an important consideration (for example, in large fields where important structures are far off‐axis), beam fluence modulation using multileaf collimators (MLC) can be used.^(^ ^4^ ^,^ ^5^ ^)^

Removal of the flattening filter from a linear accelerator permits a considerable increase in the dose rate delivery during both step‐and‐shoot intensity‐modulated radiation therapy (IMRT) and volumetric‐modulated arc therapy (VMAT).^(6)^ The first reported clinical use of a FFF beam was in 1991 to reduce the long treatment time for high‐dose radiosurgical cases.^(7)^ A variety of other treatment planning studies have since examined the implications of removal of the flattening filter for its potential to reduce beam‐on time and, consequently, patient treatment time.^(^ ^3^ ^,^ ^6^ ^,^ ^8^, ^9^, ^10^ ^)^ Mancosu et al.^(11)^ have also recently reported in detail on their institutional experiences using FFF beams in VMAT delivery configurations for liver SBRT treatments.

Recently available commercial linear accelerators have FFF beams that provide dose rates (2400 MU/min) higher than have been utilized clinically with a conventional flattening filter (300–600 MU/min) or with a modified flattening filter (800–1000 MU/min). The Varian TrueBeam (Varian Medical Systems, Palo Alto, CA) has two high‐intensity FFF modes: a 6 MV photon beam (1400 MU/min) and a 10 MV photon beam (2400 MU/min). These high‐dose rates have the potential to improve treatment efficiency for computer optimized treatment plans. In a recent study at our institution, we demonstrated that clinical use of a FFF linear accelerator for lung and liver SBRT reduces treatment delivery time by 50% compared to conventional dose rates.^(12)^

In this study we compare the beam and treatment delivery times for a variety of clinical cases including conventional, hypofractionated, and radiosurgical computer optimized plans; in particular, we focus on those in which employing flattening filter‐free beams or VMAT delivery configuration may provide an advantage and be considered for clinical study.

## II. MATERIALS AND METHODS

A variety of cases were selected for study including tumors commonly treated with intensity‐modulated radiation therapy (IMRT), volumetric‐modulated arc therapy (VMAT), and stereotactic radiation. The cases types are shown in Table 1. Planning target volumes (PTV) ranged from 2.41 to 581.23 cm^3^. Mean target volume was 175.79 cm^3^. Target prescriptions, organ‐at‐risk (OAR) dose criteria, and beam geometry were consistent with either standard clinical practice at our institution or, if applicable, appropriate RTOG protocol standards.(13) The beam arrangements for each case and each treatment delivery modality are also shown in Table 1. For instance, most patients with low‐risk prostate cancer are treated with either a five‐field DMLC (dynamic multileaf collimator) IMRT beam arrangement or a single VMAT arc. More complex plans, such as cervical cancer or head and neck cancer with regional nodal irradiation, require two arcs in most cases. For all treatment planning cases except the whole breast treatment, 6 MV non‐FFF mode was compared to four FFF modes including 6 MV DMLC IMRT, 6 MV VMAT, 10 MV DMLC IMRT, and 10 MV VMAT. The 6 MV non‐FFF mode was used as the benchmark because 10 MV non‐FFF is not clinically available on the TrueBeam. The whole breast plans utilized tangent beams with forward planned DMLC IMRT tissue compensation. VMAT plans were not generated for the breast case.

**Table 1:** The details of delivery configuration for each case and plan type used in this study, and in general, at our institution. “E‐comp” refers to electronic compensation. For multiple cranial metastases, we typically now use four nonaxial arcs

*Fraction*	*Case Type*	*Rx Dose (Gy)*	*Fraction Size (Gy)*	*6 MV flat DMLC 600 MU/min*	*6 MV FFF DMLC 1400 MU/min*	*6 MV FFF VMAT 1400 MU/min*	*10 MV FFF DMLC 2400 MU/min*	*10 MV FFF VMAT 2400 MU/min*
*Conventional*	Meningioma	54	1.8	7 field nonaxial	7 field nonaxial	*2* nonaxial arcs	7 field nonaxial	*2* nonaxial arcs
	Breast Tangents	45	1.8	Tangents w/e‐comp	Tangents w/e‐comp	n/a	Tangents w/e‐comp	n/a
	Cervix w/Elective Pelvic Nodes	50.4	1.8	7 field axial	7 field axial	2 axial arcs	7 field axial	2 axial arcs
	Lung	70	2	4 axial beams	4 axial beams	1 arc	4 axial beams	1 arc
	Prostate	70	2	5 field axial	5 field axial	1 arc	5 field axial	1 arc
	Head and Neck	70	2	9 field axial	9 field axial	2 axial arcs	9 field axial	2 axial arcs
*Hypo*	Partial breast	38.5	3.85	4 field nonaxial	4 field nonaxial	2 axial arcs	4 field nonaxial	2 axial arcs
*Radiosurgery*	Spine SBRT	16	16	7 field fan	7 field fan	2 axial arcs	7 field fan	2 axial arcs
	Triple Cranial Metastasis	18,18,&16	18,18,&16	9 field axial	9 field axial	2 axial arc	9 field axial	2 axial arcs
	Cranial Metastasis	18	18	7 field nonaxial	7 field nonaxial	3 nonaxial arcs	7 field nonaxial	3 nonaxial arcs
	Lung SBRT	60	20	13 field nonaxial	13 field nonaxial	2 axial arcs	13 field nonaxial	2 axial arcs

For each case and treatment modality, clinically acceptable plans were generated utilizing the Varian HD‐120 multileaf collimator (MLC) (Varian Medical Systems). When applicable, the appropriate RTOG protocol was referenced for acceptable planning criteria. All plans utilized a single isocenter, including the triple cranial metastases case. The treatment technique for single isocenter multiple target VMAT has been previously described.^(14)^ Plans were generated, inversely computer optimized with sliding window, and calculated in Varian Eclipse 8.9 with RapidArc (Varian Medical Systems). Version 8.9.08 was utilized for all simulation models. The field geometry details for each plan are presented in Table 2. A UAB faculty radiation oncologist reviewed the dose volume histogram (DVH), dose color wash, and isodose level contour maps for each case and ensured clinical equivalence among plan types. Criteria included target coverage, maximum dose, organ‐at‐risk dosing (e.g. $V_{20}^{\text{lung}}$), and conformity for stereotactic cases. Sample DVHs are included as 1, 2. The plans were delivered to a water‐equivalent plastic phantom. Respiratory gating was not utilized for lung cancer cases. Endpoints of the study included beam‐on time and total treatment time. The number of monitor units (MUs) and mean body dose for each plan were tabulated, as well.

**Table 2:** The geometry of each plan type by case. For the partial breast VMAT cases where partial arcs were used, the start and stop angles are given with the rotation direction

*Case*	*Plan*	*Field*	*Gantry Rotation*	*Collimator Rotation*	*Couch Rotation*
*Meningioma*		1	103	30	0
		2	52	30	0
	DMLC	3	309	30	0
	IMRT	4	206	30	0
		5	90	30	330
		6	270	30	30
		7	300	30	90
	VMAT	1	180	30	315
		2	180	330	45
*Cervical*		1	45	0	0
		2	90	0	0
	DMLC	3	135	0	0
	IMRT	4	225	0	0
		5	270	0	0
		6	315	0	0
		7	0	0	0
	VMAT	1	180	30	0
		2	180	330	0
*Prostate*		1	0	0	0
	DMLC	2	90	0	0
	IMRT	3	120	0	0
		4	240	0	0
		5	270	0	0
	VMAT	1	360	0	0
*Lung*		1	154	0	0
	DMLC	2	103	0	0
	IMRT	3	52	0	0
		4	206	0	0
	VMAT	1	360	30	0
*Head & Neck*		1	0	0	0
		2	40	0	0
		3	60	0	0
	DMLC	4	120	0	0
	IMRT	5	140	0	0
		6	240	0	0
		7	300	0	0
		8	320	0	0
		9	340	0	0
	VMAT	1	360	30	0
		2	360	330	0
		1	45	260	0
	DMLC	2	235	97	0
	IMRT	3	325	90	90
		4	40	90	90
	VMAT	1	60 CCW 180	45	0
		2	180 CW 60	315	0
*Breast Tang*.	DMLC	1	300.9	0	278.1
	IMRT	2	126.7	0	82.2
*Spine*		1	110	0	0
		2	140	0	0
		3	160	0	0
	DMLC	4	180	0	0
	IMRT	5	200	0	0
		6	220	0	0
		7	250	0	0
	VMAT	1	360	45	0
		2	360	45	0
*Triple Met*		1	0	0	0
		2	40	0	0
		3	80	0	0
	DMLC	4	120	0	0
	IMRT	5	160	0	0
		6	200	0	0
		7	240	0	0
		8	280	0	0
		9	320	0	0
	VMAT	1	360	0	45
		2	360	0	45
*Single Met*		1	230	0	0
		2	120	0	0
	DMLC	3	90	0	315
	IMRT	4	270	0	45
		5	270	0	90
		6	315	0	90
		7	0	0	90
		1	360	25	0
	VMAT	2	180	25	45
		3	180	25	315
*Lung SBRT*		1	170	90	0
		2	130	90	0
		3	50	0	0
		4	0	0	0
		5	325	90	0
	DMLC	6	270	90	0
	IMRT	7	215	0	0
		8	295	90	355
		9	90	0	350
		10	90	90	10
		11	15	0	90
		12	335	0	90
		13	190	0	90
		1	360	30	0
	VMAT	2	360	30	0

Beam‐on time was defined as the total time that the beam was engaged and delivering radiation. Treatment time included the time from start of the first beam/arc to the cessation of the last beam/arc. These data are automatically recorded by the TrueBeam delivery system, and were subsequently extracted and compiled after delivery. In conventional IMRT plans, whenever any continuous sequences of axial fields were present, automated field sequencing (AFS) was employed. AFS loads all fields into a treatment cache and allows the linac to proceed immediately to delivery of the next field without direct input from the radiation therapist. Automated field sequencing (AFS) is an important aspect of optimizing treatment efficiency when operating the linac in DMLC IMRT mode. On the TrueBeam, AFS can automate groups of up to ten coplanar fields within a 359.9° span. In general, AFS reduces the treatment time by 20–25 sec per coplanar field, and by 15–20 sec for each split‐carriage field. As with VMAT, utilizing AFS requires vigilant therapist operation, as not all types of patient movement will automatically pause the beam. At present, AFS is not available during respiratory gating on the TrueBeam.

**Figure 1:**
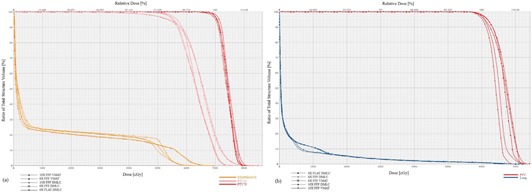
DVH's for the important structures in: (a) the head and neck case, which received 70 Gy in 35 fractions of 2 Gy; and (b) the lung SBRT case, which received 60 Gy in 3 fractions of 20 Gy. Small differences in Dmax were not judged to be clinically significant.

**Figure 2:**
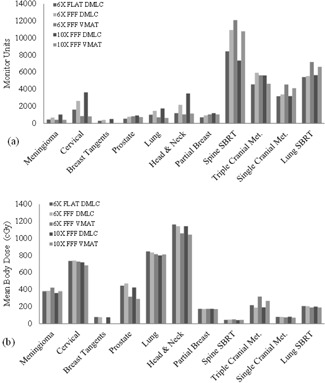
Monitor units (a) delivered for each case and plan type; mean body dose (b) for each plan.

Plans with nonaxial beam arrangements assume a room entry and a table adjustment for each noncoplanar beam which were included in the treatment time, but not beam‐on time. No time was allocated for pre‐ or intrafraction image guidance. All recorded treatment, beam‐on times, MU counts, and integral doses were statistically compared at a significance level of 0.05. For homoscedastic sample data with normally distributed residuals, we used ANOVA testing. If normality or homoscedasticity assumptions were violated, we used the Kruskal‐Wallis test, the nonparametric analogue of ANOVA.

## III. RESULTS & DISCUSSION

### A. Plan quality

Overall, we were able to achieve clinically acceptable and equivalent plan quality among all beam modes for each case scenario. Although minor differences were apparent in the DVH, all dosimetric constraints were met for each plan type. The differences in the DVH were carefully inspected by an experienced radiation oncologist and not judged to be clinically significant. Figure 1 demonstrates this with a DVH for both a conventional fractionation and SBRT plan.

Because radiation‐induced secondary cancers are always of hypothetical concern,^(15)^ we compared differences of monitor units (MUs) and integral dose to the body. The MU counts for each case and delivery configuration are shown in Fig. 2. Distributions of MU counts did not significantly differ by plan type (p=0.90). Sorting plans into conventional and hypofractionations did not alter this relationship. On an individual case basis, we did note that for the lung SBRT case, the MU count was highest for VMAT configurations, which are in agreement with the results of the analysis of Zhang et al.^(16)^ of the dosimetric properties of lung SBRT VMAT plans. Mean dose also did not vary by plan type (p=0.998).

It is germane to note here that delivery of one MU of a flattened beam requires a greater number of photons generated at the target than one MU of an unflattened beam, due to absorption and scatter by the flattening filter. As Cashmore et al.^(17)^ have pointed out, removing the flattening filter reduces scatter and associated dose to distant organs. This may indicate that each MU of an unflattened beam is associated with a lower secondary distant cancer risk than its flattened counterpart.

There were not enough cases with identical prescription doses to statistically compare MU count with target dimensions among beam modes. However, we observed that in each of the conventional fractionation prescription doses (i.e., 1.8 and 2 Gy), the cases with larger field sizes (cervical and head & neck, respectively) that required split carriage delivery exhibited higher MU counts. In both of these case types, utilizing VMAT delivery (either 10 MV or 6 MV) rather than DMLC IMRT (6 MV Flat, 6 MV FFF, or 10 MV FFF) reduced the MU count.

### B. Treatment time efficiency

**Figure 3:**
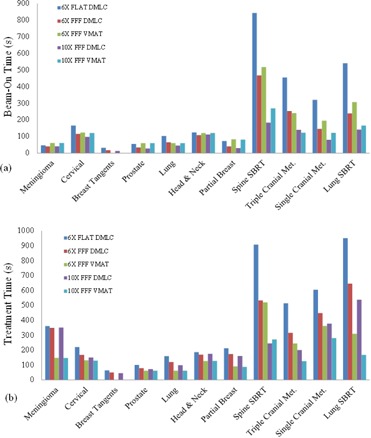
Bar graphs of: (a) beam‐on times, and (b) treatment times by case and plan type.

Clinically similar and acceptable plans were generated for each case (Table 1) for FFF and non‐FFF modes. Figure 3 shows the beam‐on and treatment times for each plan type by case. Table 3 lists the treatment efficiency improvement gained by choosing each mode instead of the 6 MV Flat DMLC IMRT beam. Treatment times for the four different flattening filter‐free plan types were compared with the flattened beam type. Utilizing FFF DMLC IMRT mode instead of the flat beam was associated with a statistically significant reduction in treatment time (p=0.005) at the 6 MV and 10 MV beam energies. Other beam delivery modes (6 MV FFF VMAT and 10 MV FFF VMAT) also reduced treatment time (p=0.005). Employing FFF mode also reduced beam time at the 6 MV and 10 MV energies (p=0.005). The 6 MV and 10 MV FFF VMAT modes reduced beam time for hypofractionated cases, but the effect was not significant for all cases (p=0.34).

**Table 3:** The relative improvement in treatment times for each delivery configuration by case type. Optimal improvements are bolded. Note that because that because beam time is gantry‐rate‐limited for conventional fractionations in VMAT, 6X and 10X FFF VMAT have essentially identical improvements for these cases

*Case Type (Fraction Size)*	*6X FFF DMLC*	*6X FFF VMAT*	*10X FFF DMLC*	*10X FFF VMAT*
Meningioma (1.8 Gy)	3.26%	58.96%	2.52%	59.23%
Cervical (1.8 Gy)	23.63%	40.42%	31.96%	41.52%
Breast Tangents (1.8 Gy)	21.97%	−	29.18%	−
Prostate (2 Gy)	22.10%	39.38%	27.38%	39.72%
Lung (2 Gy)	24.74%	61.85%	37.51%	61.80%
Head & Neck (2 Gy)	8.48%	32.24%	5.77%	31.72%
Partial Breast (3.85 Gy)	18.63%	57.66%	24.73%	59.04%
Spine SBRT (16 Gy)	41.27%	42.68%	73.03%	70.06%
Triple Cranial Metastasis (18, 18, & 16 Gy)	38.68%	52.55%	61.01%	75.55%
Cranial Metastasis (18 Gy)	25.80%	40.12%	37.51%	53.58%
Lung SBRT (20 Gy)	32.14%	67.40%	43.48%	82.35%
Mean	23.70%	49.33%	34.01%	57.46%

For small fractions delivered in VMAT mode, the dose rate is limited by the speed of the gantry rotation. In these instances, although the Varian TrueBeam is capable of delivering 2400 MU/min (or 1400 with 6 MV photons), the dose rate is reduced while the gantry's rotation velocity is maximized. Therefore, the average dose rate can be approximated by the number of MUs delivered divided by the time it takes the gantry to complete the prescribed rotation. Although the average dose rate for conventional fractionations is nearly identical for 10 MV or 6 MV VMAT delivery, the instantaneous dose rate profiles throughout the arc can vary drastically between the two.

The 10 MV flattened beam (in either DMLC IMRT or VMAT configuration) was not included for comparison. However, it is very unlikely that the 10 MV flattened beam would convey any meaningful treatment efficiency advantage relative to the 6 MV flattened beam, as it operates at the same 600 MU/min dose rate with only small differences in the depth dose distribution^(18)^ Furthermore, since all treatment modes that we investigated had equivalent plan quality, there is no reason to expect that the 10 MV flattened bean would be any different.

A reduction in treatment time without a compromise in plan quality yields a number of potential benefits including improved treatment experience for the patient, reduced likelihood of undesirable patient motion during treatment, and improved workflow for the clinic. That reduced treatment duration improves patient experience is self‐evident. An abundance of studies have demonstrated the increased likelihood of deleterious intrafraction motion (prostate^19^, ^20^, ^21^, lung^(22)^, CNS & head/neck^(23)^, gynecologic^(24)^) with increased treatment time. For example, Langen et al.^(19)^ showed the fraction of time with prostatic displacement >3mm to incrementally increase for each additional one minute interval of time. For these cases particularly, we speculate that reduced treatment time may enable reduced margins and improved plan quality. Additional work is needed to quantify the potential of improved delivery efficiency to reduce treatment margins.

The treatment efficiency improvements afforded by employing 10 MV FFF mode are meaningful to clinic workflow if treatment time slots are reduced. Although we routinely use 10 MV FFF mode for conventional fractionations, the gains in absolute time savings are small and have not significantly affected time slot scheduling. In our clinic, we schedule 15‐minute time slots for CN S and spinal radiosurgery/SBRT delivered using 10 MV FFF beams.^(12)^ Before the high‐dose‐rate FFF beams were available, we scheduled 30‐ to 60‐minute slots for these deliveries. We now allot 30‐minute time slots for FFF mode lung and liver SBRT treatments that require respiratory gating; before employing FFF mode, these slots were 60–90 min. In general, the case types with the greatest improvement in absolute treatment time savings have the most conspicuous beneficial effect on clinic workflow.

Utilizing 10 MV FFF mode and VMAT delivery yielded the greatest improvement in treatment times. The absolute treatment time improvement was greatest for hypofractionated or stereotactic cases utilizing a large dose per fraction. For example, in the lung SBRT case, treatment efficiency was improved by over 13 min ‐ a decrease of 82.4%. Treatment time improvements for all other cases are shown in Fig. 3.

For conventionally fractionated cases, utilizing unflattened beams only marginally improved treatment efficiency. This is due to the relatively low ratio of beam time to total treatment time for such cases. Absolute treatment time savings of 6 MV FFF DMLC over 6 MV non‐FFF DMLC ranged from only 11.8 to 52.1 sec are likely of minimal clinical significance. However, for cases with nonaxial fixed beam arrangements, most of the gain in treatment efficiency can still be secured by utilizing arcs which eliminate most of the table adjustments and their necessary room entries (e.g., meningioma and partial breast cases).

However, for cases in which gating or breath‐hold are viable options, even a modest beamon time reduction can enable a greater percentage of patients to maintain their inspiration for the duration of the field. Respiratory‐gated therapy is becoming more common as its ability to spare healthy lung tissue and decrease target volume is increasingly exploited.^(25)^ Although we did not include a gated treatment as one of our cases, the beam‐on per field time improvement associated with the 10 MV FFF beams may allow additional efficiency improvement by reducing the necessary number of cycles through the gating window. Treatment time savings will vary depending on which phases of the respiratory cycle are utilized, as well as the width of the gating window. Further study is needed to fully explore the benefits of more efficient FFF beam types to gated treatments. The potential benefits of deep inspiration breath‐hold in lung tumor treatment are well‐documented,^(^ ^26^ ^,^ ^27^ ^)^ and include suppressed motion, reduced margins, increased dose escalation ability, and reduction of normal lung volume receiving high dose. However, it is not employed at a very large number of treatment facilities. In a recent survey of clinical practices among users of lung SBRT techniques, only 14.4% reported using breath‐hold techniques.^(28)^ One reason for this is that it is difficult for many patients to reliably maintain proper breath hold for the time necessary to deliver the field. Because utilizing FFF mode has a large impact on beam‐on time for fixed DMLC IMRT fields, it has potential to facilitate therapy with DIBH as a viable option for previously ineligible patients. In our conventionally fractionated lung case, average time/field was reduced from 25.88 to 10.26 sec for 10 MV FFF DMLC IMRT. While verifying the value of DIBH in lung treatment, Hanley et al.^(26)^ reported that their average patient could comfortably reproduce a DIBH of 12–16 sec, ten to thirteen times per session. Therefore, in this mode, nongated uninterrupted DIBH lung therapy should be feasible for most patients.

At our institution, DIBH is utilized during delivery of breast tangents plans. The dosimetric advantages to DIBH breast therapy are also well‐established, particularly for high dose reduction to both healthy lung and cardiac tissue.^29^, ^30^, ^31^ The plans are normally delivered on our Clinac iX (Varian Medical Systems) with a wedged 6 MV flat DMLC IMRT beam. The breast tangents case presented here required 16.02 sec for each of the two fields with 6 MV non‐FFF DMLC IMRT w/electronic compensation, similar to that of our current clinical protocol. Using 6 MV FFF DMLC IMRT, delivery time for each field was reduced to 8.97 sec. Using 10 MV FFF DMLC IMRT mode with electronic compensation, the beam‐on time for each field was further reduced to 6.68 sec, a comfortably repeatable breath‐hold time for nearly every patient.

The implications of deep inspiration breath‐hold techniques have been investigated and also found to be favorable when used with partial breast irradiation.^30^, ^31^, ^32^ For the patient in our case, delivery of a single 3.85 Gy fraction required an average 18.16 sec per each of four fields in the standard 6 MV flat DMLC IMRT configuration, too long for a breath‐hold technique to be consistently implemented. When delivered in 10 MV FFF DMLC IMRT mode, average beam‐on time was just 7.76 sec per field, which also is reasonable breath‐hold expectation for nearly all patients.

In the lung SBRT case presented here, prescription was 20Gy×3 fractions, delivered via a single arc in VMAT or 13 nonaxial fields in standard DMLC IMRT arrangement. Although the total treatment time was lowest for the 10 MV FFF VMAT delivery by a wide margin, delivery within 10 MV FFF DMLC IMRT mode required the least beam‐on time; in fact, the average beam time per field was just 10.89 sec, which was nearly as short as the time required for each field in our conventional lung fractionation case. This implies feasibility of a deep inspiratory breath hold, nongated uninterrupted delivery of a 20 Gy radiosurgical fraction.

## IV. CONCLUSIONS

Use of 10 MV FFF and VMAT plan configuration yielded the greatest improvement in treatment efficiency. Efficiency gains were most pronounced in high dose per fraction cases, in particular SRS and SBRT. Although FFF and VMAT delivery modes improved treatment efficiency for conventionally fractionated plans, absolute benefits were modest and may not be clinically relevant. In clinical situations where respiratory motion management is important, 10 MV FFF mode combined with a DMLC IMRT treatment plan can be expected to deliver treatment in a single breath hold for all conventional and some stereotactic fractionations.

## ACKNOWLEDGMENTS

We would like to acknowledge Dr Kimberly Keene, Dr Robert Kim, and Dr Jennifer De Los Santos who assisted in reviewing treatment plans for clinical acceptability according their respective areas of expertise.
